# Review of COVID-19 Myocarditis in Competitive Athletes: Legitimate Concern or Fake News?

**DOI:** 10.3389/fcvm.2021.684780

**Published:** 2021-07-14

**Authors:** Zulqarnain Khan, Jonathan S. Na, Scott Jerome

**Affiliations:** ^1^Department of Medicine, University of Maryland School of Medicine, Baltimore, MD, United States; ^2^Division of Cardiovascular Medicine, Department of Medicine, University of Maryland School of Medicine, Baltimore, MD, United States

**Keywords:** COVID myocarditis, COVID-19, cardiac complications of COVID, COVID athletes, return to play, sports after COVID, pre-participation physicals, sudden cardiac death athletes

## Abstract

Since the first reported case of COVID-19 in December 2019, the global landscape has shifted toward an unrecognizable paradigm. The sports world has not been immune to these ramifications; all major sports leagues have had abbreviated seasons, fan attendance has been eradicated, and athletes have opted out of entire seasons. For these athletes, cardiovascular complications of COVID-19 are particularly concerning, as myocarditis has been implicated in a significant portion of sudden cardiac death (SCD) in athletes (up to 22%). Multiple studies have attempted to evaluate post-COVID myocarditis and develop consensus return-to-play (RTP) guidelines, which has led to conflicting information for internists and primary care doctors advising these athletes. We aim to review the pathophysiology and diagnosis of viral myocarditis, discuss the heterogeneity regarding incidence of COVID myocarditis among athletes, and summarize the current expert recommendations for RTP. The goal is to provide guidance for practitioners who will be managing and advising athletes in the COVID era.

## Introduction

In December 2019, the first case of COVID-19 caused by severe acute respiratory syndrome coronavirus 2 (SARS-CoV-2) was reported in Wuhan, China. As the global landscape has shifted to reflect the pandemic, the sports world has not been immune to these ramifications. Professional and college athletic seasons were abbreviated, fan attendance eliminated, and estimated losses of $92.6K per minute for sports occupations, along with 1.3 million jobs lost (1). While COVID infections have affected competitive athletes in similar rates to the general population, the cardiovascular implications and their ability to resume athletic participation remains unclear. Of particular concern is viral myocarditis, cardiovascular inflammation associated with a significant portion of sudden cardiac death (SCD) in athletes (ranging from 5 to 22% pre-COVID) (2). In 2020, multiple athletes opted to forgo the season due to uncertainty about returning to play following the diagnosis of COVID myocarditis, including Boston Red Sox pitcher, Eduardo Rodriguez. We will briefly review the pathophysiology and diagnosis of viral myocarditis, discuss the incidence of COVID myocarditis among athletes, and reconcile the current recommendations for return-to-play (RTP).

## Pathophysiology

Myocarditis is a nonischemic inflammatory process affecting the myocardium and inducing myocardial injury of varying clinical severity. The etiology of myocarditis may be infectious (viral, bacterial) or noninfectious (toxins, hypersensitivity, autoimmune disorders, and radiation). In viral myocarditis, which may or may not directly translate to COVID, injury to the cardiac muscle is attributable to direct virus-induced damage, as well as subsequent autoimmune inflammation. The acute phase (within hours) of viral myocarditis is comprised of viral entry into myocytes mediated by cell surface receptors (3). Once intracellular, the viral genome is translated into viral proteins, which may disrupt key dystrophin-glycoprotein interactions to impair cardiac function and injure myocyte cytoskeleton to cause myocyte death (4). During the second phase, there is an innate immune response to the viral antigen mediated by humoral (B-cell) and cell-mediated (T-cell) mechanisms. In the third phase, the host immune system may recognize intracellular components released as a result of virus-induced injury as foreign antigens, which may induce an immunologic response and autoantibodies against the myocyte (*via* CD4+ cells stimulating B-cells, cytotoxic CD8+ cells, and cytokines). Over time, these autoantigens may cause chronic myocardial inflammation, further myocyte necrosis, and progression of structural heart disease (dilated cardiomyopathy).

**Figure 1:**
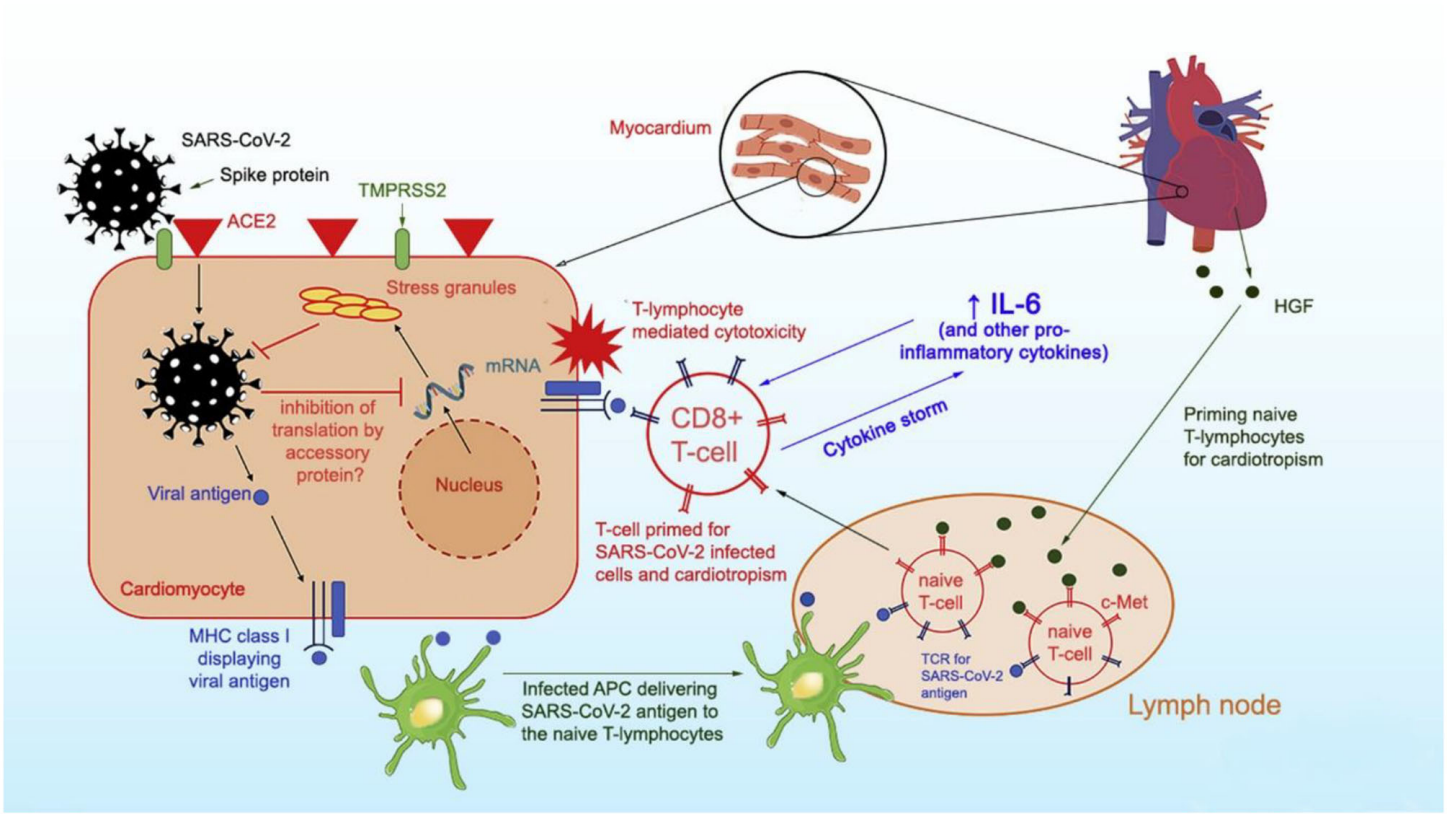
Proposed mechanism of SARS-CoV-2 entry into myocytes and inflammatory pathways causing viral myocarditis. Reprinted with permission (5).

Per Siripanthong et al. (5), the pathophysiology of COVID myocarditis is postulated to be similar with SARS-CoV-2 entering the cell by binding to angiotensin-converting enzyme 2 (ACE2) receptors on cardiomyocyte surfaces, inducing viral replication, and setting off the lymphocytic inflammatory cascade augmented by interleukin 6 (IL-6) mediated cytokine release (Figure 1). Based on this animal model, the severity of COVID associated myocarditis may reflect the immune response generated by the host, so young, otherwise healthy, athletes may generate a more robust immunologic reaction to viral infection and experience greater lymphocytic proliferation and cytokine storm.

## Diagnosis

### Clinical Presentation

The initial presentation of myocarditis is often nonspecific, so a high index of suspicion is required by the clinician. A viral prodrome (congestion, rhinorrhea, cough, and/or fever) may precede viral myocarditis. Young patients, particularly athletes, without coronary artery disease (CAD) risk factors may present with severe chest pain and ST-segment elevations on an electrocardiogram (ECG), described as an “infarct-like” pattern associated with viral myocarditis (6). Alternatively, patients may report various degrees of exertional dyspnea, atypical chest pain, palpitations, and/or generalized fatigue. In extreme cases, previously healthy patients may present with decompensated heart failure or cardiogenic shock (volume overload, depressed cardiac index, and cool extremities). The most morbid presentation is one of a patient with life-threatening arrhythmia or SCD, as a result of the nonischemic ventricular scarring induced by myocarditis, which is a nidus for re-entrant circuits (7).

### Exam

The physical exam may demonstrate subtle positional or reproducible chest pain. There may be signs of congestive heart failure, including jugular venous distension (JVD), ascites, abdominal pain, peripheral extremity edema, or crackles on a lung exam. Given the propensity for dysrhythmia, examiners should keenly evaluate for rhythm irregularities, ectopic beats, or rate discrepancies (bradyarrhythmia and tachyarrhythmia). Rarely, patients may present in fulminant cardiogenic shock as a result of COVID myocarditis with hypotension, narrow arterial pulse pressure, cool extremities, and altered mental status (8).

### Biomarkers

If viral myocarditis is suspected, clinicians should obtain markers of myocardial injury, including elevated troponin (I or T) and creatinine kinase. Elevated brain natriuretic peptide (BNP) may indicate ventricular dilation or strain from myocardial injury (9). Viral serology testing, although low sensitivity, may be reasonable if evaluating for viral myocarditis [including full respiratory viral panel, as well as SARS-CoV-2 polymerase chain reaction (PCR) testing or antibodies]. Particularly in athletes, alternative etiologies of cardiomyopathy should be excluded, such as substances (i.e., cocaine) and metabolic derangements (thyroid) with urine toxicology, and serum thyroid stimulating hormone (TSH) (10). Inflammatory markers [e.g., C-reactive protein (CRP)] can be obtained and trended with treatment.

### Electrocardiogram

In myocarditis, a 12-lead ECG may demonstrate changes such as diffuse ST-segment elevations, T-wave inversions, low-voltage QRS complexes, or even q-waves. As noted above, the infiltrative nature of viral myocarditis may ultimately result in scarring, which can impair the electrophysiological components of the heart. Even transient myocardial inflammation may induce intraventricular conduction delay, AV-block, supraventricular tachycardia (SVT), ventricular tachycardia (VT), ventricular fibrillation (VF), atrial fibrillation, or nonspecific ectopy. If inflammation extends to the pericardium, the ECG may also demonstrate PR-interval depressions (11).

### Transthoracic Echocardiography

The diagnostic workup for myocarditis should include a TTE, which can be useful in evaluating for myocarditis as well as excluding alternative etiologies of cardiomyopathy, such as valvular pathology or other structural heart disease (11). In the acute phase of viral infection, myocardial inflammation may be characterized by impaired ventricular function, abnormal ventricular dimensions (i.e., dilation or increased myocardial wall thickness), and/or pericardial effusion. Specifically in this scenario, increased wall thickness in the setting of low voltage on the ECG is suggestive of myocardial edema or infiltrative disease. Chronic myocardial inflammation may cause ventricular dilation, as well as hypokinesis, which may be global or regional (12). Although TTE findings in myocarditis can be nonspecific, specialized modalities that attempt to quantify motion of specific myocardial segments [such as strain rate imaging (SRI)] are nonstandardized and have only been utilized in case reports (13–15).

### Cardiac MRI

Given the nonspecific nature of biomarkers, symptoms, ECG, and TTE in myocarditis, CMR has been heralded as the noninvasive gold standard to evaluate myocardial inflammation, including segments not ideal for biopsy (i.e., epicardium, pericardium) (16). In 2018, the American College of Cardiology (ACC) updated the CMR diagnostic criteria for myocarditis, known as Lake Louise Criteria (LLC), to increase specificity (see Supplementary Figure 1) (12, 17). On CMR, there are three proposed diagnostic targets indicative of myocardial inflammation: myocardial edema (mediated by inflammation), hyperemia (due to increased permeability of vascular beds), and myocardial necrosis/scar (reflective of myocyte death).

According to Ferreira et al., these changes are reflected in signal intensity of various modalities within CMR imaging. Myocardial edema leads to prolonged myocardial relaxation time, which can be measured on T1 or T2 weighted images, as well as hyperintensity on T2-weighted images. An expanded extracellular space within myocardium is visualized by increased extracellular volume (ECV) or by administration of gadolinium-based contrast (GBCA), which localizes to inflamed myocardium when measured in T1 weighted imaging, known as early gadolinium enhancement (EGE). Finally, myocardial necrosis leads to scarring, which allows delayed GBCA accumulation known as late gadolinium enhancement (LGE) in T1-weighted imaging. To fulfill the updated LLC for acute myocardial inflammation (Supplementary Figure 1), CMR must identify at least one criterion of both myocardial edema (T2-based) AND nonischemic myocardial injury (T1-based). Moreover, the LLC boasts particularly high sensitivity and specificity in acute viral myocarditis, which is characterized by a CMR pattern of subepicardial edema and patchy necrosis [often at the basal inferolateral or lateral wall of the left ventricle (LV)], which may extend to mid-myocardial regions (12).

In addition to diagnostic utility, CMR also has prognostication value, per Gräni et al. In their 2017 CMR evaluation (prior to revision of LLC in 2018) of 670 suspected myocarditis patients, a 2–3 fold increase in hazard ratio was observed in development of major adverse cardiovascular events (MACE) in patients who had LGE on CMR (18). In a prognostic study more relevant for COVID myocarditis, which can present with “infarct-like” findings (positive biomarkers, ST elevations on ECG, and LGE on CMR), Chopra et al. found a greater risk of MACE compared to noninfarct-like presentations (6).

While CMR-based LLC is very accurate for diagnosis of acute inflammation, its sensitivity is reduced as myocardial inflammation becomes more diffuse. In a cost-conscious world, CMR and trained radiologists also remain cost-prohibitive for nonacademic centers.

### Endomyocardial Biopsy

The gold standard for identifying myocarditis remains endomyocardial biopsy (EMB) because it allows for histopathological, immunohistochemically, and molecular biology analysis with few complications (12, 19, 20). Given the patchy distribution of myocarditis, five or six EMB samples are recommended to reduce false negative results, but fewer may be obtained in practice (21). Histological analysis of viral myocarditis demonstrates lymphocytic infiltration of myocardium. Suspected myocardial samples can also be analyzed *via* viral nucleic acid stains and quantitative PCR or RT-PCR to evaluate for the presence of a viral genome. However, given the inherent risks of EMB (albeit cited as <1% by experienced interventionalists) and low sensitivity of obtaining affected samples, centers are more inclined to evaluate for myocarditis noninvasively with CMR and biomarkers.

## Incidence

Despite the prowess of diagnostic modalities, reported cases of COVID myocarditis have varied considerably from study to study. An in-depth evaluation reveals that earlier studies were reporting higher incidence of COVID-related myocarditis compared to those published more recently. In July 2020, Puntmann et al. (22) evaluated 100 German patients (nonathletes) recovered from COVID-19 with CMR at a median of 71 days from initial diagnosis and reported that 78% of patients had “abnormal CMR” indicative of cardiac involvement, while 60% had evidence of ongoing myocardial inflammation. These abnormal CMR findings are described as “at least one of the following” from increased myocardial T1 or T2 time, myocardial LGE, or pericardial enhancement. Interestingly, the authors do not directly reconcile their CMR findings with updated or original LLC parameters. Additionally, CMR imaging should ideally be performed in temporal proximity to the acute phase of infection but was done at a median of 71 days after COVID-19 diagnosis in the study, which makes it difficult to interpret clinical significance of the CMR changes. In September 2020, Rajpal et al. (23) published the first major study regarding COVID myocarditis in athletes from The Ohio State University. Twenty-six athletes (football, soccer, lacrosse, basketball, and track), who had PCR-confirmed COVID infection, underwent CMR, TTE, ECG, and troponin measurements following recommended quarantine (11–53 days). The published results indicate that four of these athletes (about 15%) fulfilled 2018 LLC for myocarditis with two out of those four reporting mild dyspnea, while eight others had evidence of LGE without T2 changes (23). While more expeditious than the Puntmann study, there was still latency to perform CMR in Rajpal et al., which may have failed to capture the acute inflammatory period of myocarditis in some cases. Additionally, while the incidence of myocarditis was 15%, the presence of LGE in eight athletes (which represents myocardial scarring) is certainly concerning.

In early 2021, another significant COVID myocarditis study including 145 student athletes was published by Starekova et al. (24), from the University of Wisconsin, who were recovering from COVID asymptomatically or with mild to moderate symptoms. In this elegantly designed study, these athletes underwent CMR, a median of 15 days after diagnosis, as well as measurement of biomarkers, ECG, and TTE. Of the 145 athletes, only two (1.4%) had CMR evidence of myocarditis per updated LLC, as reviewed by two experienced radiologists (24). Notably, one athlete was largely asymptomatic with mild elevation of biomarkers (troponin-I peaked at 0.09 ng/mL), while the other had mild to moderate symptoms for 3 days in the setting of normal biomarkers, and both had normal LV function. As such, the authors questioned the use of CMR as a screening tool for myocarditis in athletes without significant symptoms or abnormal ECG/biomarkers. With similar skepticism, Kawakami et al. (25) published a January 2021 pathological review with autopsy evaluation of 16 hearts (obtained from patients who had died from SARS-CoV-2) and found that only two hearts had PCR-detectable SARS-CoV-2 in the myocardium, but without pathological evidence of myocarditis. Senior author, Dr. Aloke V. Finn, noted that incidence of myocarditis with SARS-CoV-2 is lower than initially reported and cautioned that EMB be reserved for severe cases but admits that these pathological findings are mostly from older patients with co-morbidities, which do not directly translate to a younger population (i.e., athletes). Most recently, two large studies have further elucidated the prevalence of myocardial inflammation in athletes following COVID infection. In March 2021, Martinez et al. (26) evaluated 789 professional North American league athletes following COVID infection, ultimately finding that just five (0.6%) of the 789 had CMR evidence of myocarditis/pericarditis. Subsequently, Moulson et al. (27) released their findings in April 2021 that among 3,018 collegiate athletes who tested positive for COVID, 21 (0.7%) had cardiac involvement per updated LLC.

## Expert Recommendations

**Figure 2:**
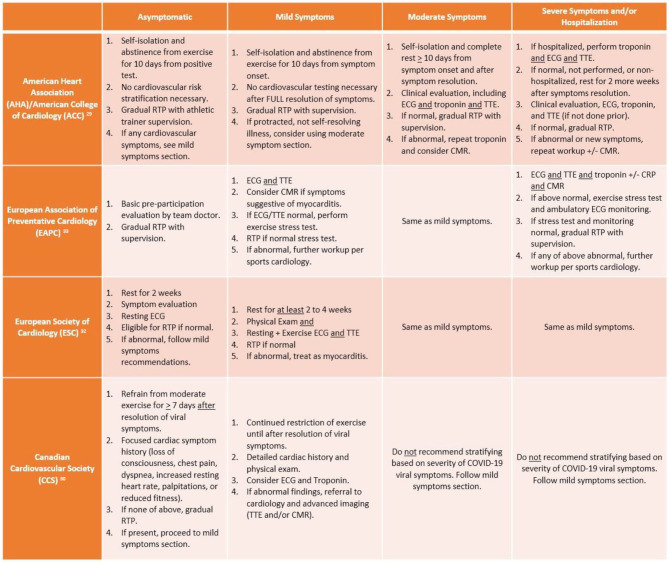
Summary of return to play (RTP) recommendations from major cardiology societies. ECG, electrocardiogram; TTE, transthoracic echocardiogram; CMR, cardiac magnetic resonance imaging; CRP, C-reactive protein.

Based on the known risk of SCD in athletes with myocarditis and aforementioned data on COVID myocarditis, various cardiology societies have attempted to generate a RTP consensus. The most up-to-date RTP recommendations for adult athletes from American, European, and Canadian societies are summarized in Figure 2. It too is worth mentioning that each society has a slight variation with respect to the isolation or convalescence period in their recommendations (e.g., 7 days to 2 weeks). While there is data extrapolated from animal models suggesting that viral replication and subsequent myocardial injury can be worsened by vigorous activity, there is no guiding data specific to the SARS-CoV-2 virus. As such, each society is basing their recommendations on epidemiologic data, which suggests that SARS-CoV-2 concentration and transmission peaks within the first week of infection, incubation lasts from 2 to 12 days, and cultivable virus is absent after 8 days (28). Taking the data into account, isolation periods ranging from 7 to 14 days seem reasonable to encompass the incubation period and allow athletes to resume their respective RTP workup.

According to the AHA/ACC, adult athletes should abstain from exercise for 10 days (or symptom resolution/no fever for 24 h) following an asymptomatic diagnosis of COVID-19 and gradually return to their previous level of activity with athletic trainer supervision. Meanwhile, per these recommendations, mildly symptomatic athletes recovering from COVID-19 do not require extensive risk stratification beyond history and physical exam if their mild symptoms were self-limited. However, in athletes with moderate to severe or not self-resolving symptoms, extensive cardiovascular risk stratification is needed, including ECG, biomarkers, and TTE (29). If testing is normal, then athletes may RTP gradually with supervision of athletic trainers, while abnormal testing or development of new cardiovascular symptoms warrants repeat biomarkers and CMR.

In contrast to the American recommendations, the European and Canadian societies are more pragmatic with RTP screening, while acknowledging the inability to offer universal cardiovascular testing in all COVID-infected athletes. Yet there are key differences between the Canadian Cardiovascular Society (CCS) and European Association of Preventative Cardiology (EAPC)/European Society of Cardiology (ESC) recommendations particularly when it pertains to COVID-symptom based stratification. According to McKinney et al., athletes should not be risk stratified based on their viral COVID illness symptoms, rather with the reporting or development of cardiovascular symptoms following recovery from acute viral illness. The CCS recommendation is based on the lack of association between severity of COVID illness and development of myocarditis, which is consistent with recent studies that have mostly identified myocarditis in asymptomatic or mildly symptomatic athletes. At that point, a cardiac symptom questionnaire should be administered; if no cardiac symptoms are reported, athletes may gradually RTP following at least 7 days of viral symptom resolution. COVID-infected athletes who report having the aforementioned cardiac symptoms require a focused history and physical exam, consideration of ECG/troponin, and referral to cardiology (for TTE and/or CMR) if any abnormal findings noted (30). Meanwhile, the EAPC and ESC advocate for use of exercise stress testing in symptomatic athletes more than the Canadian or American societies; the EAPC recommends athletes with mild to moderate symptoms should undergo ECG and TTE, then exercise stress testing for eligibility to RTP if normal, while the ESC recommends exercise ECG in tandem with TTE. However, while the ESC maintains the same recommendations for severe/hospitalization symptoms as mild to moderate cases (akin to CCS), the EAPC is more in line with the AHA/ACC in recommending a more rigorous cardiovascular evaluation consisting of imaging, biomarkers, and stress testing.

In athletes diagnosed with COVID myocarditis, the 2015 recommendations for sports eligibility by “Task Force 3” (comprised of AHA and ACC) (31) should be adapted (see Supplementary Figure 2).

## Conclusion

Early pandemic studies in nonathletes reported higher incidence of COVID-related cardiac involvement, while recent publications indicate that incidence of COVID myocarditis in adult athletes is not robust as initially feared. While the recommendations by various cardiology societies are an excellent resource, there remain limitations with regards to stratifying athletes by symptoms of viral illness. In the cited cases of athletes with CMR-proven COVID myocarditis, the affected athletes had mild to no symptoms, which means they could have been eligible for RTP without further workup per AHA/ACC and CCS guidelines (23, 24). Furthermore, at least in the Starekova et al. study, both athletes with COVID myocarditis had normal LV function, so they may have also evaded the EAPC/ESC recommendations for further workup. Nonetheless, as suggested by Moulson et al. (27), primary screening *via* CMR is also low yield unless prompted by ECG, TTE, or biomarkers. Mitigating the low prevalence of cardiac involvement in athletes with COVID with the risk of SCD, moving forward with a symptom-based approach, suggested by most societies, to guide RTP seems most appropriate.

## Author Contributions

JN and SJ contributed to the development and writing of the manuscript. All authors contributed to the article and approved the submitted version.

## Conflict of Interest

The authors declare that the research was conducted in the absence of any commercial or financial relationships that could be construed as a potential conflict of interest.
