# Supplementary material for: Review of COVID-19 Myocarditis in Competitive Athletes: Legitimate Concern or Fake News?
Source: Front Cardiovasc Med. 2021 Jul 14;8:684780. doi: 10.3389/fcvm.2021.684780 (PMC8318064; doi:10.3389/fcvm.2021.684780)
Supplement: Supplementary file 1 [file Data_Sheet_1.docx]

Supplementary Figures


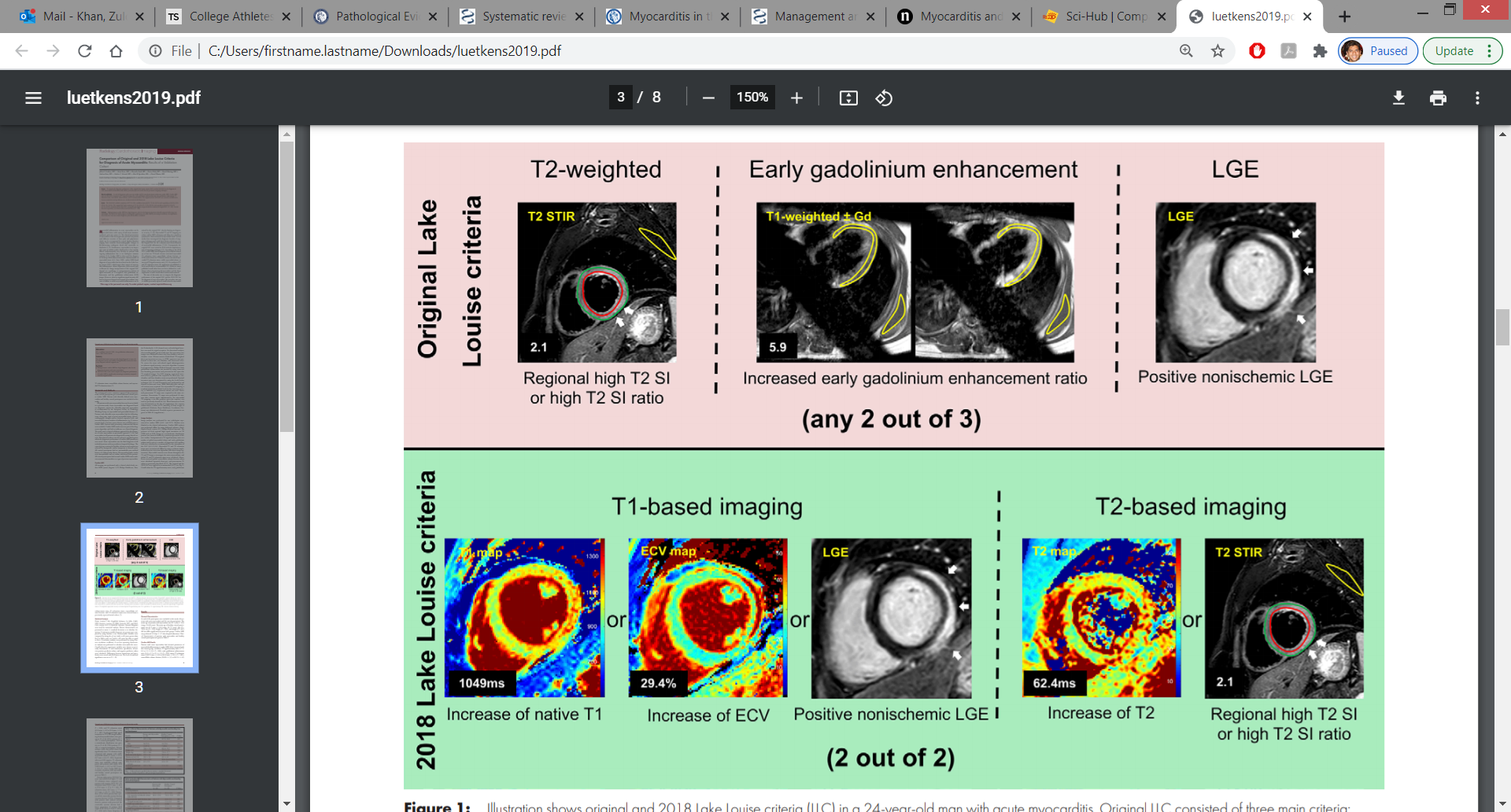


Supplementary Figure 1: Comparison of the CMR-based original and 2018 Lake Louise criteria in a 24-year-old patient with acute myocarditis. Reprinted with permission (Luetkens et al. 2019).^14^


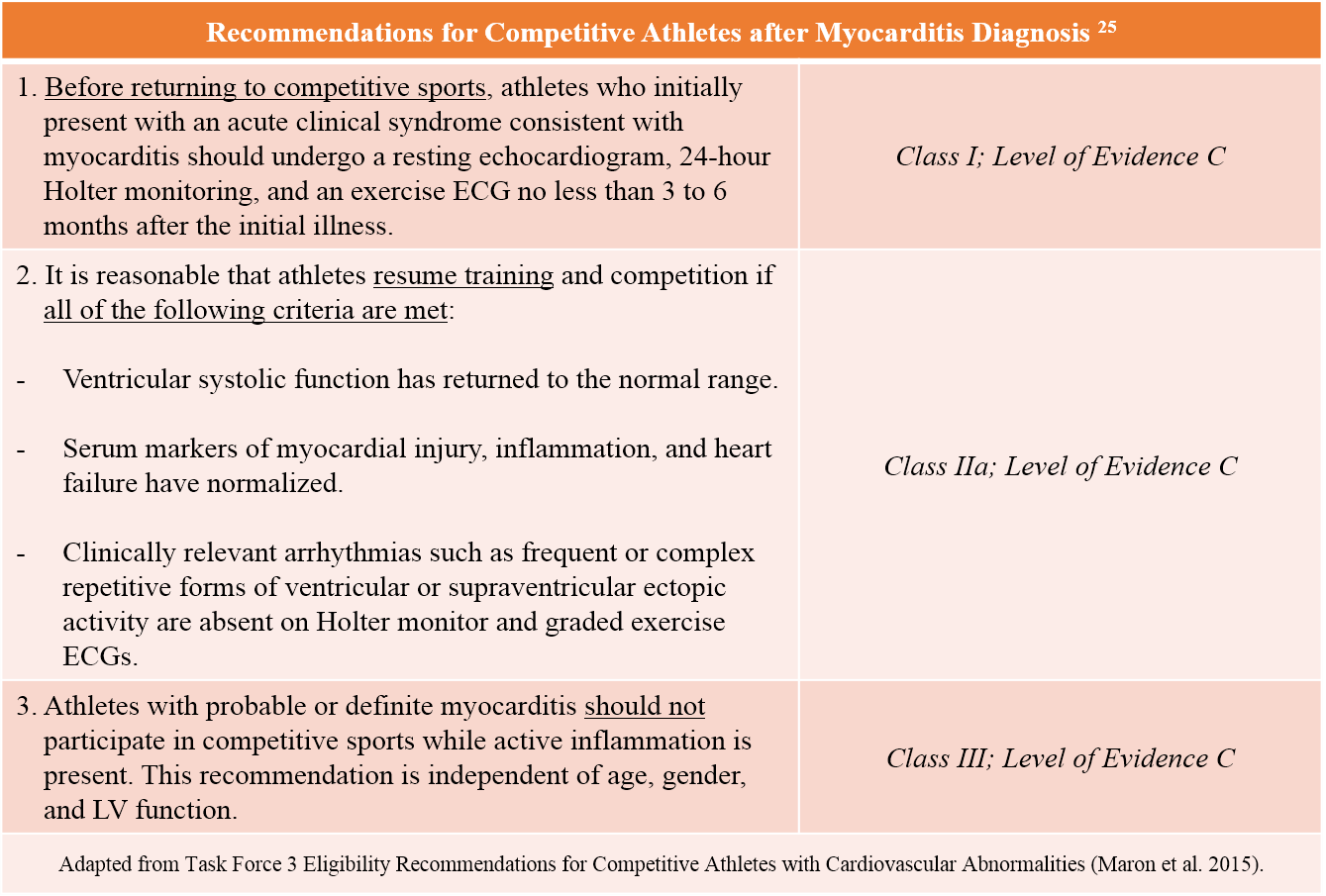


Supplementary Figure 2: Task Force 3 recommendations for sports eligibility in competitive athletes after myocarditis diagnosis.
